# Brain Fluid Clearance After Traumatic Brain Injury Measured Using Dynamic Positron Emission Tomography

**DOI:** 10.1089/neur.2024.0010

**Published:** 2024-04-22

**Authors:** Tracy Butler, Julia Schubert, Nikolaos A. Karakatsanis, Xiuyuan Hugh Wang, Ke Xi, Yeona Kang, Kewei Chen, Liangdong Zhou, Edward K. Fung, Abigail Patchell, Abhishek Jaywant, Yi Li, Gloria Chiang, Lidia Glodzik, Henry Rusinek, Mony de Leon, Federico Turkheimer, Sudhin A. Shah

**Affiliations:** ^1^ Department of Radiology, Weill Cornell Medicine, New York, New York, USA.; ^2^ Centre for Neuroimaging Sciences, King's College London, London, United Kingdom.; ^3^ Department of Mathematics, Howard University, Washington, DC, USA.; ^4^ College of Health Solutions, Arizona State University, Phoenix, Arizona, USA.; ^5^ Department of Psychiatry, Weill Cornell Medicine, New York, New York, USA.; ^6^ Department of Radiology, New York University School of Medicine, New York, New York, USA.

**Keywords:** adult brain injury, cerebrospinal fluid, clearance, glymphatic system, PET scanning

## Abstract

Brain fluid clearance by pathways including the recently described paravascular glymphatic system is a critical homeostatic mechanism by which metabolic products, toxins, and other wastes are removed from the brain. Brain fluid clearance may be especially important after traumatic brain injury (TBI), when blood, neuronal debris, inflammatory cells, and other substances can be released and/or deposited. Using a non-invasive dynamic positron emission tomography (PET) method that models the rate at which an intravenously injected radiolabeled molecule (in this case ^11^C-flumazenil) is cleared from ventricular cerebrospinal fluid (CSF), we estimated the overall efficiency of brain fluid clearance in humans who had experienced complicated-mild or moderate TBI 3–6 months before neuroimaging (*n* = 7) as compared to healthy controls (*n* = 9). While there was no significant difference in ventricular clearance between TBI subjects and controls, there was a significant group difference in dependence of ventricular clearance upon tracer delivery/blood flow to the ventricles. Specifically, in controls, ventricular clearance was highly, linearly dependent upon blood flow to the ventricle, but this relation was disrupted in TBI subjects. When accounting for blood flow and group-specific alterations in blood flow, ventricular clearance was slightly (non-significantly) increased in TBI subjects as compared to controls. Current results contrast with past studies showing reduced glymphatic function after TBI and are consistent with possible differential effects of TBI on glymphatic versus non-glymphatic clearance mechanisms. Further study using multi-modal methods capable of assessing and disentangling blood flow and different aspects of fluid clearance is needed to clarify clearance alterations after TBI.

## Introduction

Brain fluid clearance is recognized as an important homeostatic mechanism by which the brain is cleared of metabolic products, toxins, and other wastes through cerebrospinal fluid (CSF) and interstitial fluid (ISF) pathways, including the recently described glymphatic pathway.^1,2^ Clearance failure is considered a key pathophysiology underlying protein deposition in Alzheimer's Disease (AD).^3^ Brain fluid clearance may also be important in the pathophysiology of traumatic brain injury (TBI). After TBI, there is neuronal debris, inflammation, and release of proteins and toxins that need to be cleared from the brain. Given that the AD pathognomonic proteins amyloid-β (Aβ) and tau are released after TBI,^4^ failure to effectively clear them may relate to why TBI is a risk factor for AD and other dementias.^5–7^ Because glymphatic clearance is greatest during sleep,^8^ and sleep is known to be profoundly disrupted after TBI, reduced glymphatic clearance may be a mechanism by which impaired sleep contributes to poor TBI recovery.^9^ This is important because improving sleep after TBI is a feasible therapeutic target.

Animal studies suggest that failed glymphatic clearance after TBI leads to protein deposition and neurodegeneration.^4,10^ Whether brain fluid clearance is impaired after TBI in humans is only beginning to be studied. Two recent studies (one by us) demonstrate impaired glymphatic clearance after TBI using a magnetic resonance imaging (MRI) technique called diffusion tensor imaging along perivascular spaces (DTI-ALPS),^11,12^ which quantifies ISF diffusivity along periventricular medullary veins as a DTI-ALPS Index.^13^ However, DTI-ALPS has been criticized because it measures diffusivity in only a tiny portion of the brain, and also because it reflects only glymphatic diffusivity along veins, whereas there are multiple, overlapping mechanisms for fluid clearance. These include bulk flow along both veins and arteries and through the ventricular system, as well as diffusion through brain parenchyma.^1,3^

There is intense interest in identifying non-invasive neuroimaging methods to assess brain fluid clearance *in vivo* in humans.^14,15^ In addition to the DTI-ALPS MRI method noted above, MRI has been used to track the movement of gadolinium-based contrast through the brain after intravenous^16^ or intrathecal^17^ injection. Intrathecal injection has been considered the most accurate measure of glymphatic function because the rate at which contrast moves paravascularly from the subarachnoid space into the ventricular system can be directly measured in real time.^17^ One small study has shown that this rate correlates with the DTI-ALPS index.^18^ However, intrathecal contrast injection is invasive and inappropriate for routine research use. Other methods considered to index aspects of fluid flow and glymphatic function include functional MRI,^19^ which measures the pulsatility of CSF flow through the ventricular system, and phase-contrast MRI, a clinically used technique that measures CSF flow velocity, typically through the cerebral aqueduct connecting the lateral and fourth ventricles. There remains significant controversy as to the validity/accuracy of these MRI methods in measuring brain fluid clearance or the glymphatic system.^14,15^

**FIG. 1.**
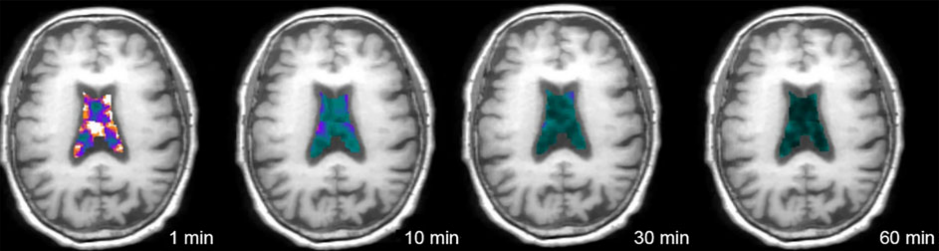
Ventricular clearance measured using dynamic PET. A single subject's PET images, superimposed over co-registered MRI, show decreasing radiotracer in lateral ventricles over the 1-h scan period. Image from Li and colleagues.^21^ MRI, magnetic resonance imaging; PET, positron emission tomography

We developed a positron emission tomography (PET) technique, termed ventricular clearance, that measures the rate at which intravenously injected radiotracer disappears (i.e., is cleared) from the ventricle.^11,20–25^ This is shown in Figure 1. This is a net measure of clearance reflecting several different but overlapping processes including directional flow of CSF within the ventricular system (including to the subarachnoid space for glymphatic clearance), CSF mixing by pulsatile back-and-forth flow and diffusion within the ventricular system, and tracer dilution by new CSF production and diffusion of ISF into the ventricle.^26–28^ Ventricular clearance can be calculated from dynamic PET using many low-molecular-weight tracers by either compartment modeling performed over the entire length of scanning^22–24^ or as the normalized rate of clearance during a defined scanning interval.^11,20,21,25^ Impaired ventricular clearance has been demonstrated in AD and in association with Aβ deposition.^11,20–22,25^

We recently demonstrated a moderate correlation between PET-measured ventricular clearance and the DTI-ALPS index, helping to cross-validate both techniques as measures of brain fluid clearance.^11^ We found that these two measures contributed independently to predicting the extent of brain Aβ deposition in healthy older subjects. Brain Aβ deposition is arguably the only clear-cut evidence of past clearance failure in humans that is currently available. That ventricular clearance and the ALPS index both contributed independently to explaining Aβ deposition, with ventricular clearance explaining a slightly greater proportion of variance, highlights that different neuroimaging modalities are sensitive to different aspects of fluid clearance. Multi-modal neuroimaging is essential for measuring and understanding brain fluid clearance in human health and disease.

Here, for the first time, we apply our PET method for measuring ventricular clearance to subjects with TBI and controls who underwent PET with the radiotracer, ^11^C-flumazenil (FMZ). FMZ binds to gamma-aminobutyric acid type A receptors expressed by neurons throughout the brain and is considered to reflect neuronal integrity.^29^ We focus not on the pattern of FMZ binding (the typical use of PET), but rather on the washout of FMZ from the ventricle (where there is no binding) as a measure of fluid clearance. We also compare ventricular clearance to (previously) MRI-measured ALPS index,^11^ considered to specifically reflect glymphatic function.^13^

## Methods

### Subjects

TBI subjects (*n* = 7) were recruited through rehabilitation and trauma departments. Control subjects (*n* = 9) were recruited through advertisements. All subjects provided informed consent, and all study activities were approved by Weill Cornell's institutional review board. This is the subset of subjects reported in our previous MRI publication^11^ who had also undergone FMZ PET. TBI subjects had sustained a complicated mild (Glasgow Coma Scale [GCS] 13–15 with intracranial lesion) or moderate-severe TBI (GCS ≤12) within the past 3–6 months. Control subjects were free of past TBI as assessed by the Brain Injury Screening Questionnaire.^30^

### Magnetic resonance imaging acquisition

T1-weighted magnetization-prepared rapid gradient echo (0.8 mm isotropic) and multi-shell diffusion-weighted MRI (1.5 mm isotropic, b = 1500, 3000; 98 directions per shell) were acquired on a 3T Siemens Prisma scanner with a 32-channel head coil (Siemens Medical Solutions USA, Malvern, PA). See Butler and colleagues^11^ for details.

### Positron emission tomography acquisition

PET data were acquired over 60 min beginning at the time of injection of 420–611 MBq of FMZ on a Siemens mCT™ PET/CT (computed tomography) scanner. All scans occurred between 12:00 pm and 4:00 pm. PET data were binned into 22 frames in a 400 × 400 matrix with a voxel size of 1.082 × 1.082 × 2.025 mm^3^. See Kang and colleagues^29^ for details.

### Positron emission tomography processing

PET images were motion corrected and linearly co-registered to MRI using FSL.^31^ Bilateral lateral ventricle and whole-brain gray matter (except cerebellum) regions of interest (ROIs) were defined using Freesurfer^32^ on MRI and transformed to PET space using the inverse transformation matrix from co-registration. ROIs were eroded 2 mm to minimize partial volume effects. FMZ time-activity curves (TACs) were extracted from each ROI for compartment modeling.

Following published methods,^33^ an image-derived input function (IDIF) reflecting blood/radiotracer delivery to the brain was extracted from bilateral 4-mm circular ROIs manually placed on summed (0–90 sec) transaxial PET images co-registered to MRI and then projected onto each frame to obtain carotid TACs. ROIs were checked on every frame for accurate placement.

### Positron emission tomography modeling

**FIG. 2.**
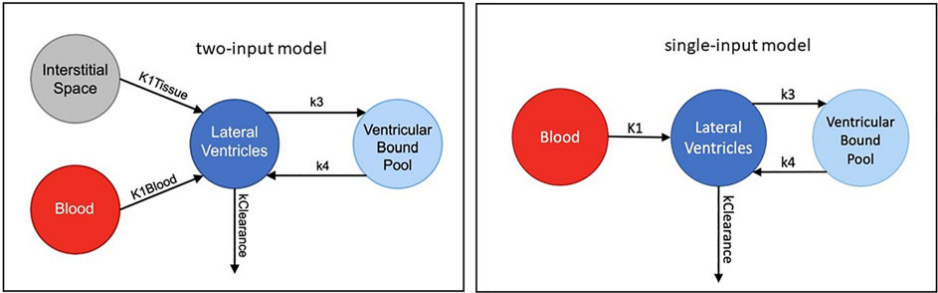
Clearance compartment models evaluated.

In accord with past studies,^22–24^ and as shown in Figure 2, we modeled ventricular clearance (k_clearance, the rate of radiotracer clearance from the ventricles) with two input functions: k1_blood reflecting tracer/blood delivery to ventricles by the choroid plexus and k1_tissue reflecting delivery of unbound tracer to ventricles through gray matter ISF.^26–28^ We also assessed model fit with only the blood input function.

SAAM II software^34^ was used to iteratively fit the ventricle TAC using the clearance compartment models. The unbound gray matter TAC (estimated by fitting a two-tissue compartment model) was used as the input from interstitial space, whereas the IDIF was used as the input from blood. Forecast standard deviation (FSD) for data-point weighting was calculated by dividing the lateral ventricle activity by the frame duration for each frame. Higher FSD values indicate lower accuracy and thus result in lower weighting. During the model fitting process, all parameters were initially adjustable and rate constants were recorded for cases where successful convergence to a single solution was achieved. These recorded rate constants were then used as priors in the model using the Bayesian estimation for all parameters. Estimated population means were calculated across control and TBI groups using a leave-one-out method, and the estimated standard deviation (SD) was set as 2*SD to provide a suitable degree of model flexibility.

### Diffusion tensor imaging along perivascular spaces

Diffusion MRI was processed as described previously to calculate the ALPS index.^25^ Briefly, the ALPS index is the ratio of diffusivity in the direction of the perivascular space and diffusivity perpendicular to both major fiber tracts and perivascular space within ROIs manually defined on color-coded, axial diffusion images. DTI-ALPS detailed methods and results in the same subjects described here have already been reported.^11^

### Statistical analysis

Analysis used SPSS software (v26; SPSS, Inc., La Jolla, CA).

Group differences in demographics and PET modeling results were assessed with a *t*-test/Wilcoxon's test for continuous variables and Fisher's exact test for categorical variables.

Multiple linear regression was performed with the dependent variable = k_clearance and key predictor = subject type (TBI vs. control). We included k1_blood in the model, reflecting tracer/blood delivery to the ventricle, given evidence of altered cerebral blood flow after TBI injury, with both increases and decreases reported.^35,36^ We also assessed age as an additional predictor. Potential interactions were checked and significant interactions retained in the model.

Spearman's test was used to compare PET-measured k_clearance to the MRI-measured ALPS index.

## Results

### Participant characteristics

Participant characteristics, including demographics and injury information, are shown in Table 1 with additional details including mechanism of injury and acute CT findings provided in the Supplementary Material. Two TBI subjects had trace intraventricular blood. TBI and control subjects did not differ significantly by age or sex (*p* > 0.1).

**Table 1.** Participant Demographics, Injury Characteristics, and Results (k_clearance and k1_blood) from PET Compartment Modeling

	***Subjects with TBI (*** *n* *** = 7)***	***Controls (*** *n* *** = 9)***
Demographics		
Sex	5 male (71.4%)	5 male (55.6%)
Mean age in years (range, sd)_	48.4 (33–58, 9.8)	51.6 (33–65, 10.6)
Injury factors		
Mean days post-injury (range, sd)	139.9 (103–213, 35.1)	N/A
Mean Glasgow Coma Scale (range, sd)	12.3 (8–15, 2.5)	N/A
Mean length of acute hospitalization in days (range, sd)	7.6 (1–24, 7.7)	N/A
Rate constants from compartment modeling		
Mean k_clearance (range, sd)	0.014 (0.007–0.020, 0.006)	0.018 (0.002–0.035, 0.011)
Mean k1_blood (range, sd)	0.004 (0.002–0.008, 0.002)	0.006 (0.002–0.010, 0.003)

Additional injury details are provided in the Supplementary Material

PET, positron emission tomography; TBI, traumatic brain injury; sd, standard deviation; N/A, not applicable

### Positron emission tomography modeling results

The two-input model with adjustable parameters and Bayesian estimation exhibited convergence issues, failing to reach a single solution in 4 of 16 cases (TBI, 3 of 7; control, 1 of 9). The simplified blood-only input model with adjustable parameters demonstrated consistent convergence to a single solution across all cases and overall had the lowest Akaike information criterion scores, signifying its superior fit to the data. The simplified model was therefore selected as the most appropriate for describing ventricular FMZ distribution. There were no significant differences in any parameters between TBI subjects and controls (all *p* > 0.1; group average values provided in Table 1), though both k_clearance and k1_blood were numerically lower in TBI subjects than controls.

### Multiple regression results

**FIG. 3.**
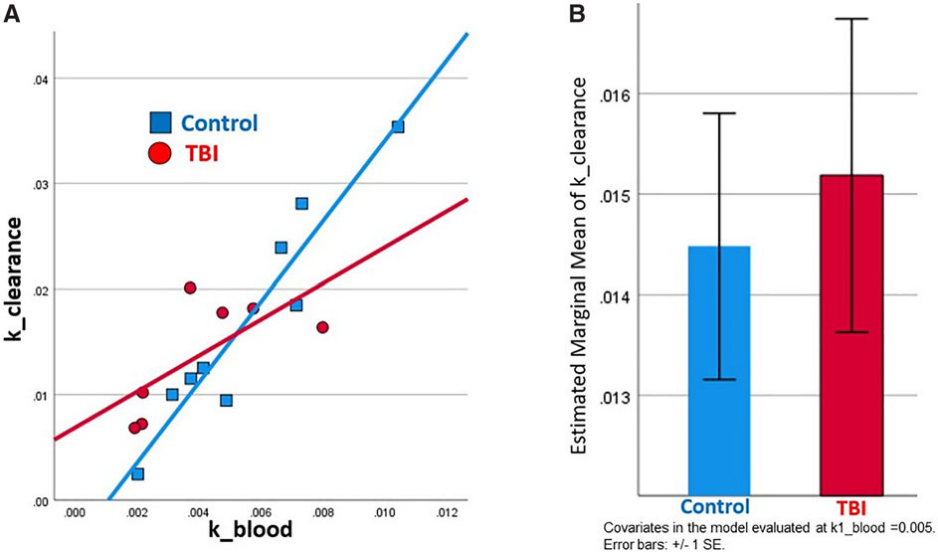
TBI versus control differences in ventricular clearance and the dependence of ventricular clearance on blood flow to the ventricle. (**A**) Plot showing the relationship between tracer/blood delivery to the ventricle (k1_blood) and ventricular clearance (k_clearance) in controls and TBI subjects. In controls, ventricular clearance is highly, linearly dependent upon tracer/blood delivery to the ventricle (*R* = 0.95, *p* < 0.001). In TBI subjects, the correlation between ventricular clearance and tracer/blood delivery to the ventricle is not significant (*R* = 0.69, *p* = 0.084). (**B**) Bar plot of estimated marginal means (controlling for k1_blood and the k1_blood × group interaction) of ventricular clearance in TBI subjects and controls. Ventricular clearance was numerically (though not significantly) greater in TBI subjects as compared to controls when accounting for these factors. SE, standard error; TBI, traumatic brain injury.

A significant regression model (*R*^2^ = 0.838, adjusted *R*^2^ = 0.798, *F* = 20.73, *p* < 0.001) showed that k1_blood (β = 5.94, *p* < 0.001), group (TBI vs. control, β = 0.011, *p* = 0.03), and their interaction (β = 2.46 = −2.43, *p* = 0.032) predicted k_clearance. The interaction between k1_blood and group was attributable to k_clearance being strongly correlated with k1_blood in controls, but not in TBI subjects, as shown in Figure 3A. Mean ventricular clearance was numerically higher in TBI subjects when accounting for tracer/blood flow delivery to the ventricle and group-specific effects on tracer/blood flow, as shown in Figure 3B. Results were similar when subject age was included as an additional predictor or when 1 control with the highest k_clearance was excluded.

### Ventricular clearance and diffusion tensor imaging along perivascular spaces correlation

FMZ PET-measured k_clearance correlated with the MRI-measured ALPS index at a trend level (*n* = 16, rho = 0.474, *p* = 0.064).

## Discussion

After TBI, clearance of blood, neuronal debris, and other substances is necessary for survival and recovery. Accurate methods for *in vivo* assessment of the efficiency and mechanisms of brain fluid clearance after TBI are essential to improve understanding of TBI pathophysiology and inform therapeutic advances. However, measuring brain fluid clearance remains challenging in humans and even in experimental animals. Here, for the first time, we applied a novel dynamic PET method to measuring brain fluid clearance after TBI. We did not detect a difference in the average rate at which intravenously administered radiotracer was cleared from brain ventricles between subjects who had experienced TBI ∼5 months earlier as compared to age-matched controls. However, we found that the correlation between ventricular clearance and blood/radiotracer delivery to the ventricle differed significantly between TBI subjects and controls. Specifically, we found that under normal conditions, ventricular clearance is highly, linearly dependent on tracer/blood delivery to ventricle. But in TBI subjects, blood flow and clearance appear to be uncoupled. We discuss the implications and limitations of these novel results.

Cerebral perfusion and vascular pulsations are considered a key driving force for glymphatic and other brain fluid clearance mechanisms.^37–39^ Our finding of a strong correlation between blood flow to ventricle and ventricular clearance in healthy subjects, though not previously demonstrated using PET, is intuitive and in accord with this previous work. Disruption of this expected correlation in TBI subjects suggests that blood/tracer delivery to ventricle is *not* the driving force for PET-measured ventricular clearance after TBI. Rather, ventricular clearance after TBI might be expected to depend upon factors such as the quantity of blood/debris that needs to be cleared, injury severity, time post-injury, and the degree of damage to different components of fluid clearance systems. TBI-induced alterations in cerebral blood flow, which are highly heterogeneous across subjects and brain regions,^35^ are also likely relevant.

We believe our results may relate to the effects of TBI on different components of fluid clearance pathways, with TBI-induced damage to some components and homeostatic upregulation of others. Reduced glymphatic clearance, attributed to aquaporin-4 mislocalization, inflammation, and other factors,^4,10^ was recently demonstrated in humans using diffusion MRI.^11,12^ We did not replicate this finding, and, in fact, ventricular clearance was slightly greater in TBI subjects when accounting for tracer/blood flow and the interaction between group and tracer/blood flow (Fig. 3B). This is not a contradiction. Ventricular clearance measured using PET reflects the net movement of fluid/tracer out of the ventricle by multiple inter-related processes—not just the glymphatic system. Current results could actually reflect compensation for TBI-induced reduced glymphatic impairment by mechanisms such as increased CSF production, which may occur after the acute TBI period.^40^

We found that PET-measured k_clearance correlated with the MRI-measured ALPS index at a trend level (rho = 0.474, *p* = 0.064), suggesting that these two methods may be measuring different but related aspects of fluid clearance. In support of this, we recently demonstrated, in a large group of normal subjects, that ventricular clearance (measured using a different PET tracer) correlated significantly with the ALPS index.^25^ With larger sample sizes, multi-modal imaging using both PET and MRI may allow assessment of the differential contribution of glymphatic versus overall fluid clearance to TBI features, including recovery.

### Limitations

The sample size for this study is quite low, and subjects were scanned relatively late (∼5 months) after injury. Larger studies that include assessment more acutely after injury are needed. Given that ventricular clearance can be measures using most radiotracers, it will be important to see whether results hold true with other tracers.

Two subjects had trace intraventricular blood on their acute CT. Although these 2 subjects did not have any evidence of overt obstruction to CSF flow at any time point (e.g., enlarging ventricles/hydrocephalus), and did not differ significantly from the other TBI subjects in any PET parameters, intraventricular blood could have subtle effects on brain fluid clearance even in the absence of overt hydrocephalus. Larger studies are needed to determine the effect of intraventricular blood on brain fluid clearance after TBI.

Glymphatic clearance is maximal during sleep,^8^ but human neuroimaging is generally performed during wakefulness. Assessing clearance during both sleep and wakefulness, though extremely challenging, could provide a more accurate estimate overall efficiency of brain fluid clearance systems. This would be especially important for understanding the interplay between post-TBI sleep impairment and clearance disruptions in TBI recovery.^9^

## Conclusion

The period post-TBI represents a critical window when the brain must be cleared of debris, inflammation, and toxins to allow recovery. There is emerging evidence in animals^4,10^ and humans^11,12^ that TBI causes dysfunction of one clearance pathway—the glymphatic system. However, measuring brain fluid clearance is challenging and it remains uncertain which components of brain fluid clearance are quantifiable using different neuroimaging methods.^1,25^ The dynamic PET ventricular clearance method used in this study measures how quickly the brain clears itself of an exogenous radiotracer molecule; this is arguably one of the most naturalistic ways to assess fluid clearance in humans. We found that under normal conditions, ventricular clearance is highly correlated with blood flow to the ventricle. After TBI, this correlation is disrupted. Although we did not detect significant group differences in ventricular clearance in this pilot study, we found that ventricular clearance was slightly (non-significantly) greater in TBI subjects when accounting for blood flow and group-specific alterations in blood flow. This contrasts with reduced MRI-measured paravascular diffusion after TBI,^11,12^ considered to reflect glymphatic function, suggesting different, potentially compensatory effects of TBI on different fluid clearance pathways and mechanisms. Results highlight the need for continued study using multi-modal methods capable of assessing and disentangling different aspects of fluid clearance after TBI.

## Authors' Contributions

T.B. conceptualized the analysis and drafted the manuscript. J.S., X.H.W., K.X., Y.K., N.A.K., K.C., L.Z., E.F., Y.L., G.C., and F.E.T. performed data analysis. S.S. conceptualized and acquired data for the larger study; all authors participated in data interpretation and reviewing and revising the manuscript.

## Funding Informartion

This work was supported by the following NIH grants: Shah: R01NS102646; Butler: R56NS111052, R01AG077576; Li: R01AG057848; de Leon: RF1AG057570, R56AG058913.

## Author Disclosure Statement

No competing financial interests exist.

## Supplementary Material

Supplementary Table S1

## Supplementary Material

Supplemental data

## Abbreviations Used

Aβ: amyloid-β
AD: Alzheimer's disease
CSF: cerebrospinal fluid
CT: computed tomography
DTI-ALPS: diffusion tensor imaging along perivascular spaces
FMZ: ^11^C-flumazenil
FSD: forecast standard deviation
GCS: Glasgow Coma Scale
IDIF: image-derived input function
ISF: interstitial fluid
MRI: magnetic resonance imaging
PET: positron emission tomography
ROIs: regions of interest
SD: standard deviation
TACs: time-activity curves
TBI: traumatic brain injury
