# Supplementary material for: Brain Fluid Clearance After Traumatic Brain Injury Measured Using Dynamic Positron Emission Tomography
Source: Neurotrauma Rep. 2024 Apr 22;5(1):359–66. doi: 10.1089/neur.2024.0010 (PMC11035850; doi:10.1089/neur.2024.0010)
Supplement: Supplemental data [file Suppl_TableS1.docx]

| Supplementary Table. TBI subject characteristics | | | | | | |
| --- | --- | --- | --- | --- | --- | --- |
| **ID** | **sex** | **age** | **injury**  **mechanism** | **acute LOS (days)** | **initial**  **GCS** | **acute CT findings** |
| 1 | M | 58 | motorcycle accident | 5 | 14 | R frontal IPH |
| 2 | M | 58 | fall from standing | 2 | 14 | bifrontal contusions and SAH |
| 3 | M | 48 | assault | 5 | 14 | SDH |
| 4 | F | 33 | fall down stairs | 9 | 9 | multifocal hemorrhage: epidural, SDH, SAH, trace intraventricular |
| 5 | M | 38 | motorcycle accident | 1 | 12 | cortical contusions; trace SAH and multiple punctate IPH c/w DAI |
| 6 | F | 49 | pedestrian struck | 7 | 15 | medial bifrontal SAH |
| 7 | M | 55 | bicycle accident | 24 | 8 | multifocal hemorrhage: SDH, SAH, trace intraventricular, punctate IPH c/w DAI |
| Abbreviations: GCS= Glasgow Coma Scale, SDH=subdural hematoma, SAH= subarachnoid hematoma, IPH= intraparenchymal hematoma, DAI=diffuse axonal injury, LOS=length of stay. | | | | | | |
